# Mechanical ventilation drives pneumococcal pneumonia into lung injury and sepsis in mice: protection by adrenomedullin

**DOI:** 10.1186/cc13830

**Published:** 2014-04-14

**Authors:** Holger C Müller-Redetzky, Daniel Will, Katharina Hellwig, Wolfgang Kummer, Thomas Tschernig, Uwe Pfeil, Renate Paddenberg, Michael D Menger, Olivia Kershaw, Achim D Gruber, Norbert Weissmann, Stefan Hippenstiel, Norbert Suttorp, Martin Witzenrath

**Affiliations:** 1 Department of Infectious Diseases and Pulmonary Medicine, Charité – Universitätsmedizin Berlin, Charitéplatz 1, 10117 Berlin, Germany; 2 Institute for Anatomy and Cell Biology, Justus-Liebig-University, Universities of Giessen and Marburg Lung Center, Aulweg 123, 35392 Giessen, Germany; 3 Member of the German Center for Lung Research, Aulweg 130, 35392 Giessen, Germany; 4 Institute of Anatomy and Cell Biology, Saarland University, Faculty of Medicine, Kirrberger Str. 100, 66424 Homburg/Saar, Germany; 5 Institute for Clinical and Experimental Surgery, Saarland University, Faculty of Medicine, Kirrberger Straße 100, 66424 Homburg/SaarHomburg, Germany; 6 Department of Veterinary Pathology, Freie Universität Berlin, Robert-von-Ostertag-Straße 15, 14163 Berlin, Germany; 7 Excellencecluster Cardio-Pulmonary System, Department of Internal Medicine, Aulweg 130, 35392 Giessen, Germany

## Abstract

**Introduction:**

Ventilator-induced lung injury (VILI) contributes to morbidity and mortality in acute respiratory distress syndrome (ARDS). Particularly pre-injured lungs are susceptible to VILI despite protective ventilation. In a previous study, the endogenous peptide adrenomedullin (AM) protected murine lungs from VILI. We hypothesized that mechanical ventilation (MV) contributes to lung injury and sepsis in pneumonia, and that AM may reduce lung injury and multiple organ failure in ventilated mice with pneumococcal pneumonia.

**Methods:**

We analyzed in mice the impact of MV in established pneumonia on lung injury, inflammation, bacterial burden, hemodynamics and extrapulmonary organ injury, and assessed the therapeutic potential of AM by starting treatment at intubation.

**Results:**

In pneumococcal pneumonia, MV increased lung permeability, and worsened lung mechanics and oxygenation failure. MV dramatically increased lung and blood cytokines but not lung leukocyte counts in pneumonia. MV induced systemic leukocytopenia and liver, gut and kidney injury in mice with pneumonia. Lung and blood bacterial burden was not affected by MV pneumonia and MV increased lung AM expression, whereas receptor activity modifying protein (RAMP) 1–3 expression was increased in pneumonia and reduced by MV. Infusion of AM protected against MV-induced lung injury (66% reduction of pulmonary permeability *p* < 0.01; prevention of pulmonary restriction) and against VILI-induced liver and gut injury in pneumonia (91% reduction of AST levels *p* < 0.05, 96% reduction of alanine aminotransaminase (ALT) levels *p* < 0.05, abrogation of histopathological changes and parenchymal apoptosis in liver and gut).

**Conclusions:**

MV paved the way for the progression of pneumonia towards ARDS and sepsis by aggravating lung injury and systemic hyperinflammation leading to liver, kidney and gut injury. AM may be a promising therapeutic option to protect against development of lung injury, sepsis and extrapulmonary organ injury in mechanically ventilated individuals with severe pneumonia.

## Introduction

In acute respiratory failure, mechanical ventilation (MV) is a life-saving intervention without alternatives, but MV may cause ventilator-induced lung injury (VILI). Since clinical trials have been highlighting the impact of VILI on acute respiratory distress syndrome (ARDS) mortality [1], lung-protective ventilation has been widely implemented in clinical practice. However, clinical and experimental studies provide evidence that VILI occurs despite low tidal volume ventilation and that particularly preinjured lungs are susceptible for the development of VILI [2,3].

Lung injury worsened in ventilated mice upon infection with *Staphylococcus aureus* even under protective ventilation strategies [4], which is intriguing as pneumonia is the leading cause of ARDS and sepsis [1,5]. However a major limitation of this and other studies was that mice were infected after initiation of MV [4,6,7]. Experimental studies investigating the impact of VILI in established pneumonia – that is, when the immune system is already activated and lung mechanics are changed due to pneumonic infiltrates – would be of particular clinical relevance.

VILI has been linked to multiple organ failure [8,9]. Improved understanding of the impact of VILI on the progression of pneumonia towards sepsis with its detrimental complications is desirable. Thus, we implemented a new second-hit model of established pneumococcal pneumonia and MV.

While the risk of ARDS development may be reduced by lowering tidal volumes, MV with low tidal volumes still seems to aggravate lung injury and further tidal volume reduction is limited by hypercapnia, which aggravates acidosis. Adjuvant pharmacotherapies in addition to protective ventilation are thus needed to further limit VILI. Adrenomedullin (AM), an endogenous 52-amino-acid peptide belonging to the calcitonin gene-related peptide family, is crucial for regulation of endothelial barrier integrity [10]. AM binds to the calcitonin receptor-like receptor (CRLR) assembled with receptor activity modifying protein (RAMP)-1 to RAMP3, thereby raising intracellular cAMP levels in endothelial cells and reducing myosin light chain phosphorylation. Consequently, interendothelial gap formation is prevented [10-12]. Exogenous AM reduced pulmonary hyperpermeability in experimental acute lung injury and sepsis [13,14], and we identified AM as being protective against VILI and associated kidney injury in previously healthy mice by stabilizing endothelial barrier function and microcirculation [13]. These and other studies gave rise to a recent positive opinion from the Committee for Orphan Medicinal Products of the European Medicines Agency (EMA), recommending the granting of the development of AM as an orphan drug for the treatment of acute lung injury/ARDS (EMA/COMP/104704/2010 to SH). However, although AM proved to be beneficial in healthy lungs subjected to VILI, evidence is lacking for a protective effect of AM during MV of individuals with severe pneumonia. Clinical trials with AM are currently being planned, so additional preclinical evidence is desirable.

We therefore conducted the current study to decipher the contribution of VILI and underlying mechanisms to the progression of ARDS, sepsis and multiple organ dysfunction syndrome in pneumonia, to test the therapeutic impact of AM in the treatment of VILI-driven lung injury in pneumonia, and to investigate potential protective effects of AM on VILI-driven extrapulmonary organ dysfunction.

## Methods

### Ethics statement

Animal experiments were approved by the animal ethics committee of Charité-Universitätsmedizin Berlin and local governmental authorities (Landesamt für Gesundheit und Soziales Berlin).

### Mice

Female C57Bl/6 mice (8 to 10 weeks; 18 to 20 g; Charles River, Sulzfeld, Germany) were used.

### Pneumococcal pneumonia

*Streptococcus pneumoniae* (serotype 3, strain NCTC7978) was grown to mid log phase. Mice were anesthetized by intraperitoneal ketamine (1.6 mg) and xylazine (0.5 mg) and were transnasally inoculated with 5× 10^6^ colony-forming units of *S. pneumoniae* diluted in 20 μl sterile phosphate-buffered saline (10 μl into each nostril) as described previously [15]. Noninfected mice were anesthetized and transnasally inoculated with 20 μl phosphate-buffered saline.

### Mechanical ventilation and adrenomedullin treatment

Twenty-four hours after infection when severe pneumonia had devolved, mice were subjected to MV as described previously [16,17]. Mice were anesthetized by intraperitoneal injections of fentanyl (75 μg/kg), midazolam (1.5 mg/kg) and medetomedin (0.75 mg/kg). Repetitively, fentanyl (16 μg/kg), midazolam (0.33 mg/kg) and medetomedin (0.16 mg/kg) were supplied via an intraperitoneal catheter when required to guarantee adequate anesthesia during the observation period. Body temperature was maintained at 37°C by a body temperature-controlled heating pad. Tracheotomy and intubation was performed, and a carotid artery catheter was placed for blood pressure monitoring and infusion of NaCl 0.9% containing 100 mmol/l HCO_3_^−^ (350 μl/hour). No additional fluid support was provided in any experiment. A urinary catheter was inserted. The tidal volume, respiratory rate, airway pressure, and urine output were monitored (Pulmodyn; Hugo-Sachs-Electronics, March-Hugstetten, Germany).

After preparation, a recruitment maneuver was performed (airway pressure 35 cmH_2_O for 5 seconds) and mice were ventilated for 6 hours with a tidal volume of 12 ml/kg, a respiratory rate of 120 breaths/minute, and a positive end-expiratory pressure of 2 cmH_2_O (MiniVent; Hugo-Sachs-Electronics). A second recruitment maneuver was performed 5 minutes before termination of the experiment. All mice survived the protocol. At termination of the experiments, mice were sacrificed by exsanguination via the carotid artery catheter. Blood samples were analyzed for partial arterial pressure of oxygen by blood gas analyzer (ABL-800; Radiometer, Copenhagen, Denmark). The P/F ratio was calculated as partial arterial pressure of oxygen/fraction of inspired oxygen. Nonventilated mice served as controls. Murine AM (0.05 mg/kg/hour; Phoenix, Burlingame, CA, USA) was continuously infused via the carotid artery catheter, starting with onset of ventilation. The dosage was proven to be effective without causing relevant hemodynamic changes in mice [13].

### Quantitative real-time polymerase chain reaction

Lungs were flushed and snap-frozen in liquid nitrogen. Total RNA was isolated from the lungs using the RNeasy mini kit (Qiagen, Hilden, Germany) according to the manufacturer’s instructions. To remove genomic DNA contamination, isolated RNA samples were treated with 1 U DNase/μg RNA (Invitrogen, Karlsruhe, Germany) for 15 minutes at 37°C. One microgram of total RNA was used in a 20 μl reaction to synthesize cDNA using Superscript H^–^ reverse transcriptase (200 U/μg RNA; Invitrogen) and oligo dTs as primers. Reverse transcription reaction was carried out for 50 minutes at 42°C. Quantitative real-time polymerase chain reaction (qRT-PCR) was performed using the I-Cycler IQ detection system (Bio-Rad, Munich, Germany) in combination with the IQ SYBR Green Real-Time PCR Supermix (Bio-Rad). The polymerase chain reaction conditions included initial denaturation in one cycle of 10 minutes at 95°C followed by 40 cycles of 20 seconds at 95°C, 20 seconds at 60°C, and 20 seconds at 72°C. The relative expressions were calculated as:

$$2^{–\left(\mathrm{\Delta CT}\right)}\times \left(1/\mathrm{mean}\;\mathrm{control}\;2^{–\left(\mathrm{\Delta CT}\right)}\right)$$

where ΔCT (CT; Threshold Cycle) is calculated as:

$$\mathrm{\Delta CT}=CT_{\mathrm{gene}\;\mathrm{of}\;\mathrm{interest}}–CT_{\mathrm{housekeeping}\;\mathrm{gene}}$$

Primer sequences are provided in Additional file 1. Regulation of CRLR and RAMP 1, 2 and 3 mRNA in uninfected, spontaneously breathing mice and uninfected, mechanically ventilated mice has been published previously [16].

### Lung permeability

Human serum albumin (HSA) was injected intravenously 90 min prior to the end of the experiment. After ligation of the left stem bronchus bronchoalveolar lavage (BAL) was performed twice with 400 μl PBS each. From each BAL portion, 250 μl were pooled and BAL and plasma HSA concentration was determined by enzyme-linked immunosorbent assay. Permeability was assessed by calculating the HSA BAL/plasma ratio as described [17].

### Hypoxic vasoconstriction in precision-cut lung slices

Precision-cut lung slices (PCLS) were prepared as described previously [18]. Briefly, mice were killed by cervical dislocation and the airways were filled with 1.5% low melting point agarose. After solidification of the agarose, the lungs were cut into 200 μm thick slices. The agarose was removed by incubation of the PCLS in phenol red-free minimal essential medium continuously gassed with 21% oxygen, 5% carbon dioxide (CO_2_), 74% nitrogen for at least 2 hours at 37°C.

To analyze the vasoreactivity of individual cross-sectioned intra-acinar arteries (minimal inner diameter up to 40 μm), the PCLS were transferred into a flow-through superfusion chamber (Hugo-Sachs-Elektronik). At the beginning of each experiment the capability of the vessel to contract in response to the thromboxane analogue U46619 and to dilate after application of the nitric oxide donor sodium nitroprusside was checked. After washing out these drugs with normoxic gassed phenol red-free minimal essential medium (21% oxygen, 5% CO_2_, 74% nitrogen), the PCLS were incubated with hypoxic gassed medium (1% oxygen, 5% CO_2_, 94% nitrogen; 0.7 ml/minute). After 15 minutes, 500 nM murine AM was added to the hypoxic medium. After a second washout, the PCLS were again challenged with U46619 in the presence of 500 nM AM when the hypoxic incubation was performed in the presence of AM or solvent, respectively.

Pictures of the artery were taken every 2 minutes using an inverted microscope mounted on the superfusion chamber. Changes in the luminal area of the vessels were evaluated by manually lining the inner boundaries. The luminal area at the beginning of hypoxia was defined as 100% and vasoreactivity was expressed as a relative decrease or increase of this area. Only the values (mean ± standard error of the mean) obtained for the hypoxic incubation followed by the incubation in hypoxic medium ± AM, the second washout phase and the final challenge with U44619 ± AM are given below.

### Leukocytes in lung tissue, bronchoalveolar lavage fluid and blood

The lungs were flushed. The left lung was digested in RPMI containing collagenase and DNAse for 1 hour. Leukocytes were extracted by meshing the lung tissue through a cell strainer (100 μm) and differentiated by flow cytometry according to their side-scatter/forward-scatter properties and CD45, Gr-1 and F4-80 expression (FACSCalibur, BD, Heidelberg, Germany). Blood leukocytes were quantified and differentiated by flow cytometry using TruCount-Tubes (BD, Heidelberg, Germany) according to cellular side-scatter/forward-scatter properties and CD45, Gr-1 and CD3 expression.

### Quantification of cytokines

Cytokines were quantified from total protein of flushed homogenized left lungs and from plasma samples by the multiplex cytokine assay technique (BioRad, Hercules, CA, USA).

### Bacterial burden

Serial dilutions of BALF, spleen homogenate and blood were plated on blood agar and incubated at 37°C under 5% CO_2_ for 24 hours to count colony-forming units.

### Creatinine, aspartate transaminase, alanine transaminase and neutrophil gelatinase-associated lipocalin

Creatinine, aspartate transaminase and alanine transaminase plasma levels were quantified by routine laboratory tests. Neutrophil gelatinase-associated lipocalin levels in urine samples collected over the last 2 hours of the MV period were measured by enzyme-linked immunosorbent assay (BioPorto, Gentofte, Denmark).

### Histology

Immunolabeling for AM was performed by overnight incubation at room temperature with previously characterized antibodies, including double-labeling with biotinylated rat monoclonal anti-CD31 (1 μg/ml, clone MEC 13.3; BD Biosciences, Heidelberg, Germany), an endothelial marker [19]. Secondary reagents, each applied for 1 hour, were Cy3-conjugated goat anti-human IgG F(ab)2 (1:500; Dianova, Hamburg, Germany), Cy3-conjugated donkey anti-rabbit IgG (1:2,000; Dianova), and fluorescein isothiocyanate-conjugated streptavidin (1:500; Sigma, Deisenhofen, Germany). Tissue sections depicting all groups were processed simultaneously and images were taken at the same exposure time.

For analysis of apoptosis, staining against cleaved caspase-3 (CC3) was used as an indicator of apoptotic cell death as described previously [20]. In brief, paraffin sections were incubated overnight with a polyclonal anti-cleaved caspase-3 antibody (Asp175, 1:50; Cell Signaling Technology, Frankfurt, Germany). A secondary antibody was added and 3,3′-diaminobenzidine served as chromogen. Apoptotic cells appeared with a brown color. The sections were counterstained with hemalaun. In each procedure, sections of thymus tissue served as positive control because apoptosis is a constant event in this organ.

### Data analyses

Data are expressed as mean ± standard error of the mean. For comparison between groups, the Mann–Whitney *U* test was used. *P* < 0.05 was considered statistically significant. For the comparison of experiments using PCLS, the area under the curve of each phase of every single experiment was calculated. Comparison between groups for each phase was performed again by Mann–Whitney *U* test, and *P* < 0.05 was considered statistically significant.

## Results

### Pulmonary expression of adrenomedullin and its receptor complexes

**Figure 1:**
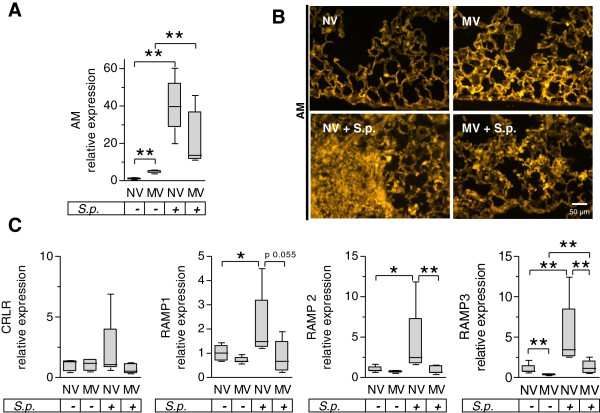
**Regulation of adrenomedullin and its receptor complexes due to pneumonia and mechanical ventilation.** Pneumococcal pneumonia (S.p.) was induced 24 hours before mechanical ventilation (MV) was performed for 6 hours. Nonventilated (NV) mice were sacrificed 30 hours after infection. Regulation of **(A)** adrenomedullin (AM) and **(C)** its receptor components calcitonin receptor-like receptor (CRLR) and receptor activating modifying protein (RAMP)- 1, 2 and 3 were quantified by quantitative real-time polymerase chain reaction in lung homogenate (*P < 0.05; **P < 0.01, *n* = 5). Note that the receptor expression data for the NV and MV groups without infection have been published previously [21]. **(B)** Immunofluorescence analysis of lung tissue. AM-immunolabeling was enhanced in MV mice compared with NV mice. In pneumonic infiltrates, leukocytes showed intense AM-immunolabeling. Representative images from five animals per group are shown.

Pneumonia and MV each increased pulmonary AM mRNA expression (Figure 1A). Immunofluorescent staining of AM in pulmonary tissue revealed that in naïve lungs AM protein was located mainly, although not exclusively, in macrophages and in pulmonary endothelium, which was confirmed by double staining of AM and the endothelial marker CD31 (see Additional file 2). In line with increased AM mRNA expression after MV, we observed a MV-induced increase of parenchymal AM protein. Furthermore, pneumonic infiltrates were positive for AM immunostaining, with recruited leukocytes displaying marked immunoreactivity (Figure 1B). AM specificity of the employed antibody was validated in pre-absorption experiments (see Additional file 3). Regulation of the AM receptor components CRLR and RAMP1 to RAMP3 was investigated by qRT-PCR analyses. As reported previously [16], MV alone had no impact on CRLR or RAMP1 and RAMP2 expression while RAMP3 was downregulated. Pneumonia resulted in an increase of RAMP1 to RAMP3 expression, while MV markedly reduced mRNA levels of RAMP1 to RAMP3 in pneumonia (Figure 1C). Notably, treatment with AM did not alter expression of AM, CRLR or RAMP1 to RAMP3 in pneumonia and subsequent MV (see Additional file 4).

Regulation of the AM receptor components CRLR and RAMP1 to RAMP3 was investigated by qRT-PCR analyses. As reported previously [21], MV alone had no impact on CRLR or RAMP1 and RAMP2 expression while RAMP3 was downregulated. Pneumonia resulted in an increase of RAMP1 to RAMP3 expression, while MV markedly reduced mRNA levels of RAMP1 to RAMP3 in pneumonia (Figure 1C). Notably, treatment with AM did not alter expression of AM, CRLR or RAMP1 to RAMP3 in pneumonia and subsequent MV (see Additional file 4).

### Mechanical ventilation exacerbated lung injury in pneumonia: protection by adrenomedullin

**Figure 2:**
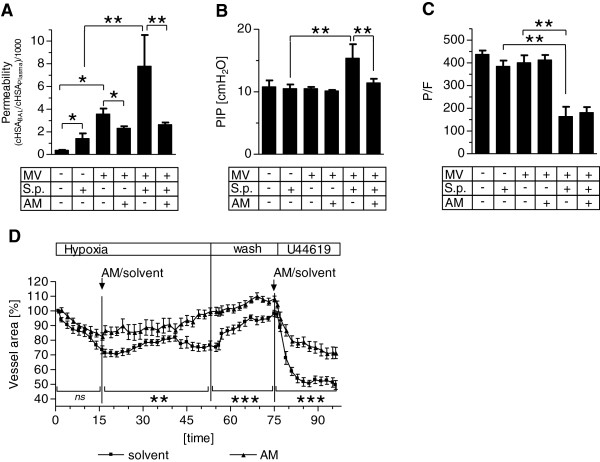
**Adrenomedullin protected mice with pneumonia against mechanical ventilation-induced lung injury.** Pneumococcal pneumonia (S.p.) was induced 24 hours before mechanical ventilation (MV) was performed for 6 hours. Continuous adrenomedullin (AM) infusion (0.05 mg/kg/hour) started with the onset of MV. Nonventilated (NV) mice were sacrificed 30 hours after infection. **(A)** Human serum albumin (HSA) was injected 90 minutes prior to termination of the experiment and the HSA concentration (cHSA) in plasma and in bronchoalveolar lavage (BAL) was determined. An increased HSA BAL/plasma ratio indicated enhanced lung permeability (*P < 0.05, **P < 0.01; NV, *n* = 5; NV + S.p., *n* = 4; MV, *n* = 5; MV + AM, *n* = 4; MV + S.p., *n* = 7; MV + S.p. + AM, *n* = 5). **(B)** Peak inspiratory pressure (PIP) was analyzed after 6 hours of MV following a final recruitment maneuver (**P < 0.01; NV and NV + S.p., *n* = 5; all other groups, *n* = 8 each). **(C)** Arterial oxygen partial pressure was determined at the end of the experiment and the P/F ratio was calculated (**P < 0.01; NV and NV + S.p., *n* = 5; MV, *n* = 6; MV + AM, *n* = 7; MV + S.p., *n* = 5; MV + S.p. + AM, *n* = 8). **(D)** In precision cut lung slices, vasoconstriction was induced by hypoxia (hypoxic pulmonary vasoconstriction) and by the thromboxane receptor agonist U46619 in the presence or absence of AM (0.5 μM). The luminal areas of single intra-acinar pulmonary arteries were continuously analyzed by planimetry and the area under the curve (AUC) was calculated for each experiment. Significant AUC differences between the two groups were evident under hypoxia, during the wash out phase and after U46619 challenge (ns, not significant; **P < 0.01, ***P < 0.001; *n* = 10 each).

Pneumonia as well as MV each increased pulmonary vascular permeability. AM reduced MV-evoked lung permeability. Notably, when mice with pneumonia were subjected to MV a further dramatic increase in lung permeability was observed, which was almost completely avoided by AM treatment starting with onset of MV (Figure 2A).

Under volume-controlled MV, an increase in the peak inspiratory pressure reflects a decrease of lung compliance, which is mostly due to lung edema in the current model. While pneumonia and MV alone had no impact on peak inspiratory pressure as compared with healthy mice, MV in infected mice led to a significant increase of peak inspiratory pressure after 6 hours of MV, which was almost completely impeded by AM treatment (Figure 2B).

While oxygenation capacity was not impaired due to pneumonia or MV alone, the combination of pneumonia and MV led towards severe deterioration of oxygenation. Although AM reduced lung injury in mechanically ventilated mice, AM did not ameliorate the deterioration of oxygenation (Figure 2C). Histology performed 24 hours post infection confirmed severe necrotizing bronchopneumonia affecting 40 to 60% of the lung tissue. Changes due to MV could not be dissected from the already prevalent severe alteration in the lungs due to pneumonia (see Additional file 5). With regard to the missing improvement 1in oxygenation despite barrier-stabilizing properties of AM and preserved lung mechanics in the pneumonia + MV group, we hypothesized that vasodilatory properties in the lung of AM might have been counteracting the reduction of lung injury by increasing ventilation/perfusion mismatch. Indeed, vasoconstriction due to hypoxia or the thromboxane agonist U46619 was significantly reduced by AM in murine PCLS (Figure 2D).

### Mechanical ventilation aggravated pulmonary inflammation in pneumonia

**Figure 3:**
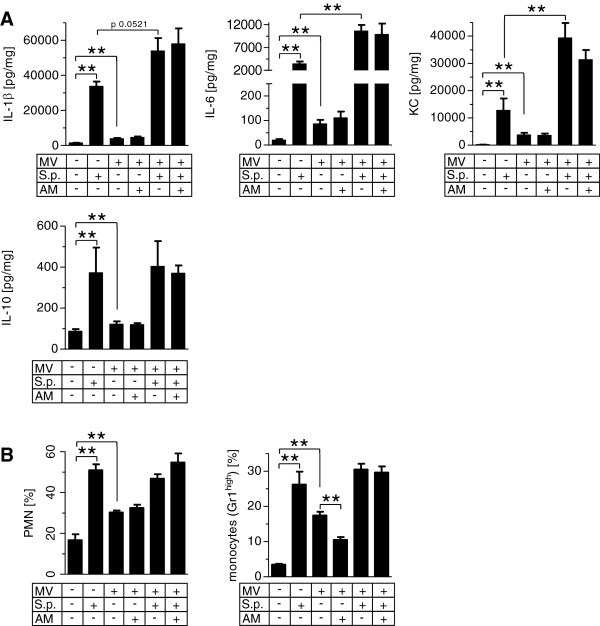
**Mechanical ventilation induced exacerbation of pulmonary inflammation in pneumonia independently of leukocyte counts.** Pneumococcal pneumonia (S.p.) was induced 24 hours before mechanical ventilation (MV) was performed for 6 hours. Continuous adrenomedullin (AM) infusion (0.05 mg/kg/hour) started with the onset of MV. Nonventilated mice were sacrificed 30 hours after infection. **(A)** Cytokine levels of interleukin (IL)-1β, IL-6, keratinocyte-derived cytokine (KC) and IL-10 were measured in lung homogenate. **P < 0.01; *n* = 6 each. **(B)** Leukocytes were isolated from lung homogenate and differentiated by flow cytometry. **P < 0.01; *n* = 5 each. PMN, polymorphonuclear neutrophils.

The concentrations of the cytokines interleukin (IL)-1β, IL-6, keratinocyte-derived cytokine (KC) and IL-10 in lung homogenate were increased by pneumonia and, to a lesser extent, by MV. In pneumonia, MV led to a further dramatic increase of IL-1β, IL-6 and KC, while IL-10 levels remained unaffected. AM treatment had no impact on pulmonary cytokine levels (Figure 3A).

In pneumonia and in MV, pulmonary polymorphonuclear neutrophils (PMN) and Gr-1^high^ monocytes were increased. A combination of MV and pneumonia did not further increase PMN and Gr-1^high^ monocytes. Notably, AM decreased pulmonary Gr-1^high^ monocyte recruitment in uninfected mice subjected to MV, but not in mice with pneumonia subjected to MV (Figure 3B).

### Mechanical ventilation had no impact on pulmonary bacterial outgrowth and development of bacteremia in pneumonia

**Figure 4:**
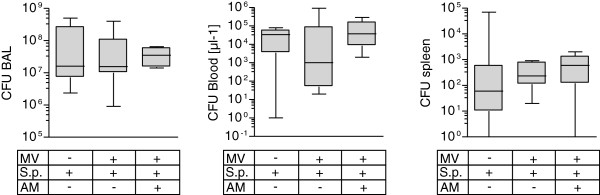
**Mechanical ventilation has no impact on lung bacterial outgrowth and systemic bacterial dissemination.** Pneumococcal pneumonia (S.p.) was induced 24 hours before mechanical ventilation (MV) was performed for 6 hours. Continuous adrenomedullin (AM) infusion (0.05 mg/kg/hour) started with the onset of MV. Nonventilated mice were sacrificed 30 hours after infection. Serial dilutions of bronchoalveolar lavage (BAL) fluid, spleen homogenate and blood were plated on agar, and colony-forming units (CFU) were counted after 24 hours of incubation (*n* = 5 to 7).

Blood and spleen homogenate bacterial counts were assessed in BALF. MV and AM each had no impact on pulmonary bacterial burden, bacteremia or dissemination to the spleen in pneumonia (Figure 4).

### Mechanical ventilation aggravated systemic hyperinflammation in pneumonia

**Figure 5:**
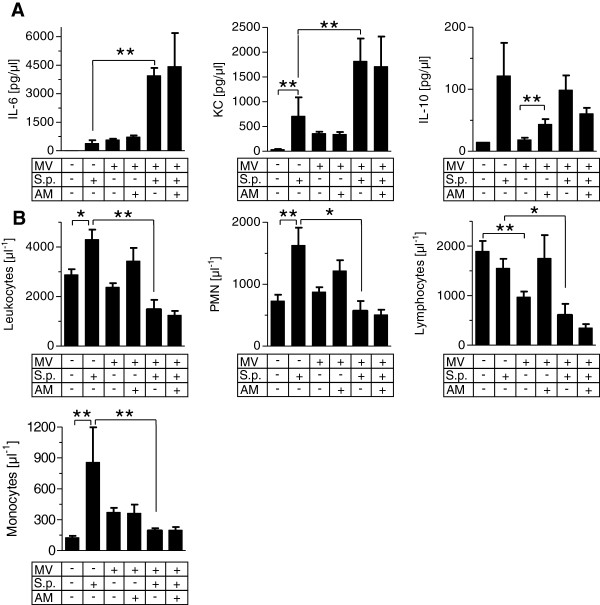
**Mechanical ventilation induced systemic hyperinflammation in pneumonia.** Pneumococcal pneumonia (S.p.) was induced 24 hours before mechanical ventilation (MV) was performed for 6 hours. Continuous adrenomedullin (AM) infusion (0.05 mg/kg/hour) started with the onset of MV. Nonventilated mice were sacrificed 30 hours after infection. **(A)** Cytokine levels of IL-6, keratinocyte-derived cytokine (KC) and IL-10 were measured in blood plasma **P < 0.01; *n* = 5 or 6 each). **(B)** Whole blood leukocytes were quantified and differentiated by flow cytometry (*P < 0.05, **P < 0.01; *n* = 5 each). PMN, polymorphonuclear neutrophils.

Pneumonia and MV each increased plasma IL-6, KC and IL-10 levels. In pneumonia, MV caused a further increase of systemic cytokine levels. AM treatment had no impact on cytokine levels (Figure 5A).

### Mechanical ventilation induced leukopenia in pneumonia

Pneumonia increased circulating neutrophils and monocytes while lymphocyte counts remained unaffected. MV reduced lymphocytes but had no effect on other leukocyte populations. In infected mice subjected to MV, blood leukocyte counts were significantly reduced compared with nonventilated mice with pneumonia, and lymphocyte counts dropped significantly below those observed in naïve mice. Besides almost restoring lymphocyte levels in uninfected mice subjected to MV, AM treatment had no significant impact on blood leukocyte counts (Figure 5B).

### Adrenomedullin protected mice with pneumonia against MV-related organ failure

**Figure 6:**
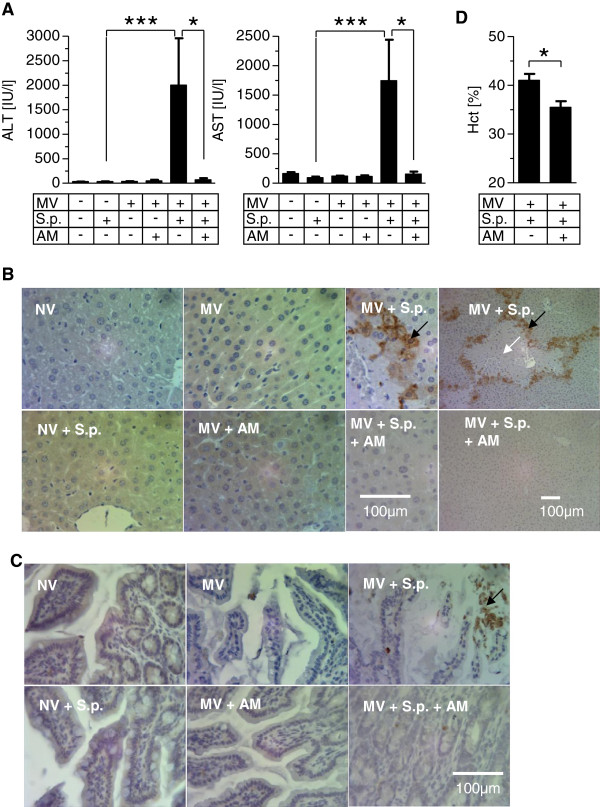
**Adrenomedullin protected against mechanical ventilation-induced liver and gut injury in mice with pneumonia.** Pneumococcal pneumonia (S.p.) was induced 24 hours before mechanical ventilation (MV) was performed for 6 hours. Continuous adrenomedullin (AM) infusion (0.05 mg/kg/hour) started with the onset of MV. Nonventilated (NV) mice were sacrificed 30 hours after infection. **(A)** Liver transaminases (alanine aminotransaminase (ALT), aspartate aminotransferase (AST)) were quantified in blood plasma (*P < 0.05, ***P < 0.001; *n* = 6 to 8 each). Liver and ileum sections were stained for cleaved caspase 3 (CC3) and counterstained with hemalaun. **(B)** In the MV + S.p. group, liver lobular necrosis (white arrow) surrounded by rings of CC3^+^ apoptotic cells (black arrow) developed, which was absent under AM treatment (MV + S.p. + AM). **(C)** Apoptotic CC3^+^ cells were observed in ileum sections of the MV + S.p. group, and to a lesser extent in the MV + S.p. + AM group. Representative images from six animals per group are shown. **(D)** Hematocrit (Hct) was reduced following AM treatment in ventilated mice with pneumonia (MV + S.p. + AM) as compared with sham-treated mice (MV + S.p.) (*P < 0.05; *n* = 5 or 6 each).

Aspartate aminotransferase and alanine aminotransaminase levels were not altered by pneumonia or MV, whereas MV in pneumonia dramatically increased transaminase levels, which was almost completely avoided by AM (Figure 6A). We observed extended liver injury displayed by necrotic areas and induction of hepatic apoptosis exclusively in the pneumonia + MV group, which was undetectable under AM treatment. More specifically, periportal rings of CC3^+^ cells were highly prominent (Figure 6B) in four out of six livers from the pneumonia + MV group and showed weaker prominence in two livers. In the liver sections of the pneumonia + MV + AM group only small, single islets of CC3^+^ cells were found. In all other groups, CC3^+^ cells were rare (0 to 5 per section). In ileum sections, an enhanced number of CC3^+^ cells was almost exclusively observed in the pneumonia + MV group, indicating tissue injury that was abolished by AM treatment (Figure 6C).

In the pneumonia + MV group increased urine neutrophil gelatinase-associated lipocalin levels were measured (see Figure S5A in Additional file 6). Pneumonia and MV each increased blood creatinine levels in comparison with naïve mice and a trend towards further increased creatinine levels in the pneumonia + MV group was observed (see Figure S5B in Additional file 6). Urine output was not altered (see Figure S5C in Additional file 6). These findings suggest early tubular injury due to MV in pneumonia unaffected by AM.

Notably, hematocrit in the pneumonia + MV + AM group was lower compared with the pneumonia + MV group, suggesting intravasal plasma conservation due to systemic stabilization of vascular barrier function by AM (Figure 6D).

## Discussion

In the current experimental study, MV induced lung injury in pneumonia and promoted sepsis and multiple organ injury. AM infusion protected against lung edema and liver and gut injury without interfering with inflammatory host responses.

For this study, we launched a novel experimental model to display the relevant interaction of VILI and pre-established pneumonia regarding lung injury, systemic inflammation and multiple organ dysfunction. VILI was induced by ventilating mice for 6 hours with moderately injurious tidal volumes of 12 ml/kg. Although 6 ml/kg is recommended for lung-protective ventilation in humans, the currently applied settings meet the requirements for protective ventilation in mice. The tidal volume of 12 ml/kg with a respiratory rate of 120 breaths/minute induced only a minor increase in lung permeability and inflammation, while having no impact on hepatic or renal injury in healthy mice [17]. Lower tidal volumes would require higher respiratory rates to ensure CO_2_ removal, which independently contributed to VILI [21] and was therefore avoided.

We first investigated the expression of AM and its receptor complexes and observed pulmonary upregulation of AM in each of both MV and pneumonia. MV increased AM expression mainly in endothelial cells and macrophages, while AM expression in pneumonia could mainly be attributed to invading leukocytes forming pulmonary infiltrates. However, MV tended to downregulate overall AM expression in pneumonia. AM binds to CRLR assembled with RAMPs, mainly RAMP2 and RAMP3 [10]. Expression of all three RAMPs was upregulated in pneumonia, and reduced by additional MV. In summary, reduced expression of RAMP1 to RAMP3 under MV in pneumonia was observed, suggesting weakened protection of endothelial integrity due to reduced endogenous AM function. Notably, AM therapy had no influence on the expression of AM, CRLR or RAMP1 to RAMP3.

We reported previously that exogenous AM protected mice against VILI even in the context of hyperoxia and delayed onset of treatment [13]. However, the applied models did not reproduce the clinically relevant situation when patients with respiratory failure due to pneumonia need MV. In the current study, pneumonia-induced lung injury was exacerbated by MV as displayed by increased permeability and edema and decreased oxygenation capacity. These changes could not be attributed to further pulmonary leukocyte recruitment due to MV, but were paralleled by a dramatic increase of pulmonary inflammatory cytokines. Some studies reported immunomodulating effects of AM offering protection against lipopolysaccharide-induced systemic hyperinflammation and lung injury or against polymicrobial sepsis in mice [22,23]. In line with these studies, pulmonary Gr-1^high^ monocytes, which have specifically been linked to VILI [24], were reduced by AM in mechanically ventilated mice without pneumonia. However, AM did not alter cytokine levels, overall leukocyte counts or Gr-1^high^ monocytes in the lungs of mechanically ventilated mice with pneumonia, suggesting that protection by AM was not primarily mediated by immunomodulation in this study but targets a central mechanism downstream of harmful hyperinflammation.

Taking the current and previous findings into account, stabilization of endothelial integrity was most probably the main mechanism of the observed protective AM effect [11,12,14,25,26]. From the clinical point of view it is preferable to stabilize lung barrier function independently from the inflammatory state because hyperinflammation is frequently established when it comes to intubation in pneumonia and sepsis, because hyperinflammation may override anti-inflammatory properties of pharmacologic therapies, and because pharmacologic immunosuppression may pave the way for secondary infections [27]. Further, while disturbance of microcirculation is a hallmark of organ failure during septic shock, AM stabilized microcirculation in inflammation [26,28]. Although not directly evidenced by the current study, this protective function of AM possibly contributed to the observed effects.

Oxygenation was not improved in AM-treated mice despite marked reduction of permeability and edema. This contradictory finding may be explained by vasodilatory AM effects. More specifically, vasoconstriction caused by the thromboxane agonist U44619 or hypoxia were diminished by AM in lung tissue slices. Although direct evidence is not provided, it is tempting to speculate that reduction of pulmonary vascular resistance by AM resulted in increased shunt perfusion, probably masking improvement of oxygenation capacity following reduction of edema formation in AM-treated mice. Importantly, AM treatment did not result in deterioration of mean systemic arterial blood pressure or microcirculatory impairment as assessed by blood lactate.

Cyclic stretch of alveolar epithelial cells in MV has been observed to enhance bacterial growth and bacteremia, thereby augmenting the development of sepsis and organ failure [6,23,29,30]. However, this mainly holds true for Gram-negative bacteria, while bacterial growth and translocation of Gram-positive bacteria such as *S. aureus* was not influenced by cyclic stretch *in vitro* or by MV *in vivo*[4,30]. Further, Gram-positive *S. pneumoniae* actively invades lung tissue, so that MV-associated bacterial translocation may be of inferior relevance particularly in pneumococcal pneumonia [31]. In the current study, all infected mice were bacteriemic and MV did not impact pulmonary *S. pneumoniae* outgrowth or bacteremia. Nevertheless, infected mice subjected to MV developed severe sepsis, whereas spontaneously breathing mice did not. Severe sepsis was displayed by dramatically increased blood cytokines, leukopenia and hepatic, renal and intestinal injury, which, most notably, could not be attributed to hemodynamic deterioration.

AM protected against hepatic and intestinal injury in VILI-driven sepsis. These findings support previous studies in which AM protected from liver or gut injury in staphylococcal α-toxin induced shock, in polymicrobial sepsis or in gut ischemia and reperfusion [14,22,32]. Again, in the majority of studies, tissue-protective effects of AM have been attributed to anti-inflammatory properties, whereas anti-inflammatory effects of AM were not detected in the current study. Apoptosis may be crucial for the development of organ failure in sepsis, and AM holds anti-apoptotic properties [33]. However, whether protection from apoptosis observed here and elsewhere is the mechanism of or a consequence of other yet unknown underlying AM functions remains unclear. The lower hematocrit in AM-treated mice currently observed indicated intravascular plasma conservation due to systemic vascular barrier protection by AM, confirming previous studies demonstrating barrier protection in the liver, ileum and kidney as a central beneficial mechanism of AM in shock [14]. Taken together, AM protected against hepatic and intestinal injury accompanied by anti-apoptotic and barrier protective effects without modulating hyperinflammation in pneumonia-associated VILI-driven sepsis. Further characterization of this potent protective AM function downstream of injurious hyperinflammation warrants further investigation.

One limitation of this study was that AM treatment could not be investigated in nonventilated mice because this requires vascular catheterization for continuous infusion due to the very short half-life of AM. This catheterization is not feasible in awake mice, and anesthesia in spontaneously breathing mice placed in a supine position for instrumentation probably results in hypoventilation, thereby provoking major bias to the sensitive readout performed here.

## Conclusion

**Figure 7:**
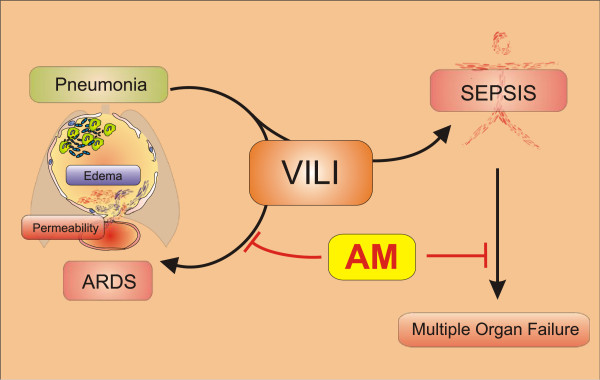
**Mechanical ventilation drives pneumococcal pneumonia into lung injury and sepsis: protection by adrenomedullin.** Ventilator-induced lung injury (VILI) led to substantial aggravation of previously established severe pneumonia resulting in acute respiratory failure, which was accompanied by elevation of pulmonary cytokine levels independent from leukocyte recruitment or bacterial replication in the lung. Adrenomedullin (AM) protected against development of lung injury without having anti-inflammatory or antimicrobial functions, probably by mediating vascular barrier protection and stabilization of microcirculation. VILI induced the development of sepsis and related multiple organ failure, which was avoided by AM. ARDS, acute respiratory distress syndrome.

This study provides evidence that MV with moderate tidal volumes aggravates lung injury and promotes progression of sepsis and multiple organ failure in pneumococcal pneumonia. Exogenous AM, which has gained orphan drug status from the EMA for treatment of ARDS recently, protected against MV-induced lung injury and sepsis-related organ failure without suppression of the host immune response (Figure 7). These data further encourage current efforts to evaluate AM as adjuvant therapy for VILI in addition to protective ventilation strategies and for sepsis-related organ failure in clinical trials.

## Key messages

• In mice with pneumococcal pneumonia, MV evoked lung injury and led to the development of sepsis with multiple organ injury independent of bacterial translocation.

• AM infusion protected against lung injury and extrapulmonary organ failure in this condition.

• Being acknowledged as an orphan drug for ARDS treatment by the EMA, AM therapy may be a potential future adjuvant pharmacotherapy in patients with severe pneumonia subjected to MV.

## Abbreviations

AM: Adrenomedullin; ARDS: Acute respiratory distress syndrome; BAL: Bronchoalveolar lavage; BALF: Bronchoalveolar lavage fluid; CC3: Cleaved caspase 3; CO2: Carbon dioxide; CRLR: Calcitonin receptor-like receptor; CT: Threshold cycle; EMA: European Medicines Agency; HSA: Human serum albumin; IL: Interleukin; KC: Keratinocyte-derived cytokine; MV: Mechanical ventilation; PCLS: Precision-cut lung slices; PMN: Polymorphonuclear neutrophils; qRT-PCR: Quantitative real-time polymerase chain reaction; RAMP: Receptor activity modifying protein; RPMI: Roswell Park Memorial Institute medium; VILI: Ventilator-induced lung injury.

## Competing interests

The authors declare that they have no competing interests.

## Authors’ contributions

HCM-R and MW planned and supervised the study, performed *in vivo* experiments, analyzed the data and drafted the manuscript. DW and KH performed animal experiments, flow cytometry and cytokine assays, and critically revised the manuscript for important intellectual content, WK, MDM, TT, OK and ADG performed histology and critically revised the manuscript for important intellectual content. RP performed experiments using PCLS and critically revised the manuscript for important intellectual content. UP performed qRT-PCR analyses and critically revised the manuscript for important intellectual content. SH, NW and NS were involved in the study design and participated in drafting the manuscript. All authors read and approved the final version of the manuscript.

## Supplementary Material

Additional file 1: Table S1 Presenting the used primer sequences for qRT-PCR. Click here for file

Additional file 2: Figure S1 Showing the pulmonary distribution of AM. Click here for file

Additional file 3: Figure S2 Showing the specificity of the used AM antibody. Click here for file

Additional file 4: Figure S3 Showing gene expression of AM, CRLR and RAMP1 to RAMP3 under treatment with AM during MV in pneumonia. Click here for file

Additional file 5: Figure S4 Showing histologic lung pathology of pneumonia before induction of MV and after the ventilation period. Click here for file

Additional file 6: Figure S5 Showing that MV induced tubular injury in pneumonia (neutrophil gelatinase-associated lipocalin levels in urine, creatinine levels in blood, urine output). Click here for file
