# Supplementary material for: Mechanical ventilation drives pneumococcal pneumonia into lung injury and sepsis in mice: protection by adrenomedullin
Source: Crit Care. 2014 Apr 14;18(2):R73. doi: 10.1186/cc13830 (PMC4056010; doi:10.1186/cc13830)
Supplement: Additional file 1: Table S1 — Presenting the used primer sequences for qRT-PCR. [file cc13830-S1.docx]

**Table 1**

Primer used for RT-PCR

---------------------------------------------------------------------------------------------------------------

gene sequence product accession

length number

---------------------------------------------------------------------------------------------------------------

AM forward GAAGCCCACATTCGTGTCA 138 bp NM009627

reverse TGCCGTCCTTGTCTTTGTC (434-572)

β-actin forward GTGGGAATGGGTCAGAAGG 299 bp NM007393

reverse GGCATACAGGGACAGCACA (212-511)

CRLR forward GCAGGACCCCATTCAACA 185 bp AF209905

reverse GGATGCCGAAACCAGTGT (169-345)

RAMP1 forward ATGGTGTGACTGGGGAAAGA 205 bp NM031645

reverse CAATGAAAGGGCAGAGGATG (205-410)

RAMP2 forward TCCCTGAACCAATCTCTTCC 185 bp NM019444

reverse GTCGCTGTAATGCCTGCTAA (260-445)

RAMP3 forward GCAACGAGACAGGGATGC 312 bp BC024765

reverse GCCACAGTCAGCACGACA (116-428)

----------------------------------------------------------------------------------------------------------------
