# Supplementary material for: Mechanical ventilation drives pneumococcal pneumonia into lung injury and sepsis in mice: protection by adrenomedullin
Source: Crit Care. 2014 Apr 14;18(2):R73. doi: 10.1186/cc13830 (PMC4056010; doi:10.1186/cc13830)
Supplement: Additional file 2: Figure S1 — Showing the pulmonary distribution of AM. [file cc13830-S2.docx]

**Additional Figure 1**

**Additional Fig 1. Pulmonary distribution of Adrenomedullin**

Adrenomedullin (AM) immunolabelling colocalized with CD31-immunoreactivity a marker for endothelial cells. In addition, alveolar macrophages were AM positive. (n = 5, representative images shown).
