# Supplementary material for: Mechanical ventilation drives pneumococcal pneumonia into lung injury and sepsis in mice: protection by adrenomedullin
Source: Crit Care. 2014 Apr 14;18(2):R73. doi: 10.1186/cc13830 (PMC4056010; doi:10.1186/cc13830)
Supplement: Additional file 3: Figure S2 — Showing the specificity of the used AM antibody. [file cc13830-S3.docx]

**Additional Figure 2**

**Additional Fig 2. Specificity of the used Adrenomedullin antibody**

Preabsorption of the Adrenomedullin (AM) antibody with mouse AM resulted in almost complete absence of labelling, suggesting high specificity of the primary antibody. Both tissue sections were cut from the same specimen and processed simultaneously. Images were taken at the same exposure time (190 ms).
