# Supplementary material for: Mechanical ventilation drives pneumococcal pneumonia into lung injury and sepsis in mice: protection by adrenomedullin
Source: Crit Care. 2014 Apr 14;18(2):R73. doi: 10.1186/cc13830 (PMC4056010; doi:10.1186/cc13830)
Supplement: Additional file 4: Figure S3 — Showing gene expression of AM, CRLR and RAMP1 to RAMP3 under treatment with AM during MV in pneumonia. [file cc13830-S4.docx]

**Additional Figure 3**

**Additional Fig 3. Regulation of AM, CRLR, RAMP 1-3 under treatment with AM during mechanical ventilation in pneumonia.**

Pneumococcal pneumonia (S.p.) was induced 24h before mechanical ventilation (MV) was performed for 6h. Non-ventilated individuals (NV) were sacrificed 30h after infection. Regulation of AM and its receptor components calcitonin receptor like receptor (CRLR) and receptor activating modifying proteins (RAMP) 1-3 were quantified by qRT-PCR in lung homogenate ( n=4).
