# Supplementary material for: Mechanical ventilation drives pneumococcal pneumonia into lung injury and sepsis in mice: protection by adrenomedullin
Source: Crit Care. 2014 Apr 14;18(2):R73. doi: 10.1186/cc13830 (PMC4056010; doi:10.1186/cc13830)
Supplement: Additional file 5: Figure S4 — Showing histologic lung pathology of pneumonia before induction of MV and after the ventilation period. [file cc13830-S5.docx]

**Additional Figure 4**


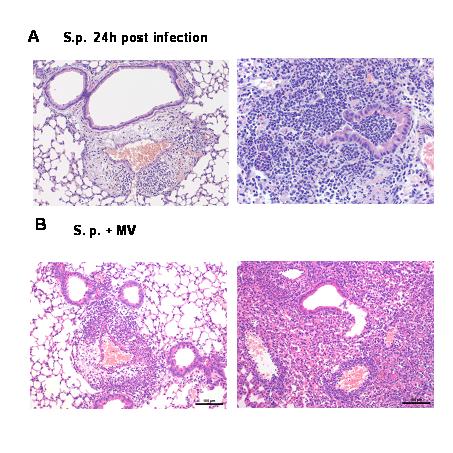


**Additional Fig 4. Histologic lung pathology of pneumonia**

Pneumococcal pneumonia (S.p.) was induced 24h before mechanical ventilation (MV) was performed for 6h.

(A) Images from mice 24h after infection with s.pneumoniae are shown, that display severe necrotizing bronchopneumonia affecting 40-60% of the lung. (B) Images from mice subjected to MV for 6h 24h after infection with S. pneumoniae. Again severe necrotizing bronchopneumonia was found. No changes due to MV could be dissected from the pneumonia induced severe damage of the lung parenchyma. Representative images out of 4 animals per group are shown.
