# Supplementary material for: Mechanical ventilation drives pneumococcal pneumonia into lung injury and sepsis in mice: protection by adrenomedullin
Source: Crit Care. 2014 Apr 14;18(2):R73. doi: 10.1186/cc13830 (PMC4056010; doi:10.1186/cc13830)
Supplement: Additional file 6: Figure S5 — Showing that MV induced tubular injury in pneumonia (neutrophil gelatinase-associated lipocalin levels in urine, creatinine levels in blood, urine output). [file cc13830-S6.docx]

**Additional Figure 5**

**Additional Fig 5. Mechanical ventilation induced tubular injury in pneumonia**

Pneumococcal pneumonia (S.p.) was induced 24h before mechanical ventilation (MV) was performed for 6h. Continuous AM infusion (0.05 mg/kg/h) started with the onset of MV. Non-ventilated individuals (NV) were sacrificed 30h after infection.

(A) Neutrophil gelatinase-associated lipocalin (NGAL) levels were quantified by ELISA in urine samples collected during the last 2h of the MV experiments. (B) Creatinine levels were determined in blood plasma (n.d. not detected, *p<0.05, ***p<0.001; n=6-8 each) (C) Urine output during the last 2h of mechanical ventilation (n=8 each).
